# Multiple pathways of SARS-CoV-2 nosocomial transmission uncovered by integrated genomic and epidemiological analyses during the second wave of the COVID-19 pandemic in the UK

**DOI:** 10.3389/fcimb.2022.1066390

**Published:** 2023-01-20

**Authors:** Kate F. Cook, Angela H. Beckett, Sharon Glaysher, Salman Goudarzi, Christopher Fearn, Katie F. Loveson, Scott Elliott, Sarah Wyllie, Allyson Lloyd, Kelly Bicknell, Sally Lumley, Anoop J. Chauhan, Samuel C. Robson, The COVID-19 Genomics UK (COG-UK) consortium

**Affiliations:** ^1^ School of Pharmacy and Biomedical Science, University of Portsmouth, Portsmouth, United Kingdom; ^2^ School of Biological Science, University of Portsmouth, Portsmouth, United Kingdom; ^3^ Centre for Enzyme Innovation, University of Portsmouth, Portsmouth, United Kingdom; ^4^ Portsmouth Hospitals University NHS Trust, Portsmouth, United Kingdom

**Keywords:** COVID-19, SARS-CoV-2, nosocomial infection, hospital-acquired infection, transmission dynamics, alpha variant, Oxford Nanopore Technologies (ONT), whole genome sequencing (WGS)

## Abstract

**Introduction:**

Throughout the global COVID-19 pandemic, nosocomial transmission has represented a major concern for healthcare settings and has accounted for many infections diagnosed within hospitals. As restrictions ease and novel variants continue to spread, it is important to uncover the specific pathways by which nosocomial outbreaks occur to understand the most suitable transmission control strategies for the future.

**Methods:**

In this investigation, SARS-CoV-2 genome sequences obtained from 694 healthcare workers and 1,181 patients were analyzed at a large acute NHS hospital in the UK between September 2020 and May 2021. These viral genomic data were combined with epidemiological data to uncover transmission routes within the hospital. We also investigated the effects of the introduction of the highly transmissible variant of concern (VOC), Alpha, over this period, as well as the effects of the national vaccination program on SARS-CoV-2 infection in the hospital.

**Results:**

Our results show that infections of all variants within the hospital increased as community prevalence of Alpha increased, resulting in several outbreaks and super-spreader events. Nosocomial infections were enriched amongst older and more vulnerable patients more likely to be in hospital for longer periods but had no impact on disease severity. Infections appeared to be transmitted most regularly from patient to patient and from patients to HCWs. In contrast, infections from HCWs to patients appeared rare, highlighting the benefits of PPE in infection control. The introduction of the vaccine at this time also reduced infections amongst HCWs by over four-times.

**Discussion:**

These analyses have highlighted the importance of control measures such as regular testing, rapid lateral flow testing alongside polymerase chain reaction (PCR) testing, isolation of positive patients in the emergency department (where possible), and physical distancing of patient beds on hospital wards to minimize nosocomial transmission of infectious diseases such as COVID-19.

## 1 Introduction

The COVID-19 pandemic marks the most significant infectious disease outbreak event that has been monitored in near real-time by whole genome sequencing (WGS). To date, there have been more than 10 million SARS-CoV-2 genome sequences submitted to the Global Initiative for Sharing of All Influenza Data (GISAID) database worldwide (GISAID - Submission Tracker Global., 2021). A large proportion of these were initially submitted by the United Kingdom (UK completes over one million SARS-CoV-2 whole genome sequences - GOV.UK., 2021), with approximately 25% of all GISAID sequences originating from the UK (GISAID - Submission Tracker Global., 2021), which can be largely attributed to the national genomic surveillance program of the COVID-19 Genomics UK (COG-UK) Consortium (COVID-19 Genomics UK (COG-UK), COVID-19 Genomics UK Consortium., 2021). Such large-scale WGS programs have enabled the identification of biologically distinct variants, including variants of concern (VOCs), classified by the World Health Authority (WHO). More than 1,700 variants and sub-variants have been named to date (based on the Pango Lineage definitions, which will be used throughout), of which five have been classified as VOCs (Cov-Lineages., 2021). In addition, genomic data have been used to inform the governmental implementation of non-pharmaceutical interventions, such as national lockdown measures, to prevent viral transmission. These datasets have also been used to investigate SARS-CoV-2 transmission in hospitals and other healthcare settings, both as a rapid-turnaround service to inform infection control when an outbreak occurs (Blackstone et al., 2021; Stirrup et al., 2021; Stirrup et al., 2022) and on a retrospective basis to understand the dynamics of nosocomial transmission at a larger scale (Illingworth et al., 2021; Lumley et al., 2021; Snell et al., 2022).

With more than 515 million cases and 6 million deaths recorded worldwide (WHO Coronavirus (COVID-19) Dashboard | WHO Coronavirus (COVID-19) Dashboard With Vaccination Data., 2021), COVID-19 represents a severe threat to public health. SARS-CoV-2 has an incubation period of up to 14 days before symptom onset (Jiang et al., 2020), with only a subset of individuals going on to display symptoms. This observation makes asymptomatic and pre-symptomatic transmission a significant concern for COVID-19 infection control (Day, 2020; Yu and Yang, 2020), which is particularly true in clinical settings and long-term care facilities where patients are physically close to each other and healthcare workers (HCW) (Huff and Singh, 2020). Visitors to healthcare settings may also act as vectors for viral transmission from the community or between patients and HCW in different localities. COVID-19 acquired in the clinical setting has a severe impact on clinically vulnerable patients (Bhogal et al., 2021) and up to 20% of infections in inpatients and 73% in HCWs may be due to nosocomial transmission in the UK (Evans et al., 2021).

Understanding the dynamics associated with nosocomial transmission will provide useful insights for infection control within hospitals for the current pandemic and future outbreaks of a similar nature. Epidemiological data relating to patient location and timing between admission and testing positive are commonly used to determine whether infections are hospital-acquired or not (COVID-19: epidemiological definitions of outbreaks and clusters in particular settings - GOV.UK., 2021). Whilst this information is useful, it cannot be used to disentangle complex transmission networks or networks involving HCWs who are in and out of the hospital setting every day. WGS of SARS-CoV-2 and subsequent phylogenetic analyses offer the possibility of elucidating related cases based on sequence similarity, providing some temporal and directional indication of transmission chains.

The second wave of the pandemic in the UK, which occurred between September 2020 and May 2021, placed significant strain on the healthcare service due to the high numbers of COVID-19 infections and admissions to hospital. Two events occurred at the peak of this wave in December 2020 that may have affected the transmission dynamics of SARS-CoV-2 within hospital settings – the commencement of the national vaccination program and the emergence of the VOC Alpha, B.1.1.7. The B.1.1.7 variant is characterized by several mutations, including the N501Y mutation on the spike protein, that increases the binding affinity of the virus to human ACE-2 receptors, thus conferring greater potential for infection (Tian et al., 2021). However, serological testing revealed that B.1.1.7 did not increase the risk of infection in vaccinated individuals (Planas et al., 2021). It was also predicted not to significantly affect nosocomial transmission compared to other variants (Boshier et al., 2021). Large-scale genomic data can also be used retrospectively to validate these findings, providing insights into both vaccine efficacy and the appearance of the B.1.1.7 Alpha variant on the transmission dynamics of SARS-CoV-2 within healthcare settings.

In this large-scale investigation, we performed Nanopore sequencing on SARS-CoV-2 samples from HCWs and patients at Queen Alexandra Hospital (QAH), Portsmouth, UK. SARS-CoV-2 sequences from 694 HCWs and 1,181 patients were combined with epidemiological information and were analyzed in detail to understand transmission dynamics within the hospital during the second wave of the COVID-19 pandemic. The aims were to identify nosocomial outbreaks within the hospital, disentangle transmission networks, compare these between variants and transmission clusters and understand the effects of HCW vaccination on SARS-CoV-2 prevalence. As the world begins to recover from the impact of the COVID-19 pandemic, we must use results from such studies to fully understand the most significant paths of nosocomial infection and help to impact policies for future outbreaks of pathogens. These analyses highlighted the difficulties faced by the hospital during the most challenging months of the COVID-19 pandemic and the power of genomic epidemiology for understanding outbreaks in detail.

## 2 Materials and methods

### 2.1 Study site and Infection Prevention & Control (IPC) procedures

Portsmouth Hospitals University NHS Trust (PHU) is one of England’s largest acute hospital trusts. Queen Alexandra Hospital (QAH) is a research hospital within the trust with an 800-bed capacity and treats > 500,000 patients a year. During the peak of the second wave of COVID-19 in the UK, 60% of hospital beds were occupied by SARS-CoV-2 positive patients.

Throughout the course of the pandemic, key actions were taken by the Infection Prevention & Control (IPC) Team at PHU to help limit COVID-19 infections within clinical settings. A timeline of these key Trust actions can be seen in **Supplementary Figure 1**. Key actions include: the required usage of personal protective equipment (PPE) for all HCWs from March 2020, to protect staff and patients from onward nosocomial viral spread; mandatory requirement of face coverings for all individuals on site from 22^nd^ June 2020, including visitors and outpatients; initiation of the staff vaccination program (which commenced on the 10^th^ December 2020), providing early access to the Pfizer/BioNTech BNT162b2 vaccine to HCWs at PHU; introduction of point of care testing (POCT) in the emergency department (introduced on the 8^th^ February 2021) which allowed patients to be cohorted from admission; and introduction of a “management of patients” pathway in May 2020, which was regularly updated based on national guidance.

This pathway map helped to simplify the safe movement of patients into and across the Trust during the pandemic. **Supplementary Figure 2** shows the guidance used by PHU at the end of the study period in May 2021, which identified specific “green” (free from COVID-19) and “red” (COVID-19 treatment) wards for protection of vulnerable patients. Surgical wards, as well as renal, haemotology and oncology wards, were protected as “green” wards. Patients underwent rapid PCR (see Section 2.2 below) on admission, and were isolated or moved to the medical bed base pending results. Elderly care wards have historically been located in older infrastructure of the hospital (circa 1970s), which rely on natural ventilation only, with air purification systems introduced to areas with poor ventilation. However, following updates to the patient pathway, infectious elderly patients were additionally moved to wards in the new estate (circa 2009), which had access to improved mechanical ventilation facilities.

### 2.2 Laboratory diagnosis

COVID-19 tests for hospital staff and patients and members of the local community within Portsmouth and surrounding areas were carried out at the Clinical Microbiology Department at QAH. Samples were collected from participants using nasopharyngeal swabs and stored and transported in Sigma-Virocult 3mL Viral Transport Media (VTM) (Medical Wire & Equipment, Corsham, UK).

The majority of testing over the period of this study was performed on the Panther system with the Aptima SARS-CoV-2 assay (Hologic, Marlborough, USA). Capacity was approximately 600 tests per day. Prior to testing, 500μL of VTM was added to a Panther Specimen Lysis Tube (Hologic, Marlborough, USA). This method involves automated RNA extraction and transcription-mediated amplification, providing a qualitative result to confirm the presence or absence of SARS-CoV-2 by amplifying two conserved regions of the SARS-CoV-2 ORF1ab gene, comparing the fluorescence signal to an internal control.

POCT was performed using the ID Now SARS-CoV-2 assay (Abbott Laboratories, Chicago, USA). Urgent testing within the laboratory was performed directly on samples in VTM using the Xpert^®^ Xpress SARS-CoV-2 assay on the GeneXpert (Cepheid, California, USA), as per the manufacturer’s instructions for use. This is a cartridge-based system for rapid detection, extraction and amplification using real time (RT)-PCR to detect 2 targets for SARS-COV-2 in the N2 and E gene regions, alongside internal controls. As urgent testing was a finite resource, less than 4% of testing was performed on this platform.

Surge capacity was provided using the Anatolia Geneworks SARS-CoV-2 PCR v2, which has 2 SARS-CoV-2 targets: ORF1ab and E gene alongside an internal control. VTM sample extraction was performed on the QIAsymphony SP/AS extraction system (Qiagen, Hilden, Germany) off-board lysis protocol (PATHOGEN, COMPLEX 200_OBL_V4_DSP) using the QIAsymphony DSP Virus/Pathogen Midi or Mini Kit and RT-PCR amplification was performed on the LightCycler4800 II (Roche, Basel, Switzerland).

### 2.3 Sampling

COVID-19-positive swab samples identified in the period between 1^st^ September 2020 and 31^st^ May 2021 were targeted for viral extraction and whole-genome sequencing. Samples from PHU, along with cases from a wide range of NHS Trusts across the South Coast of the UK, were sequenced by the University of Portsmouth as part of the COG-UK consortium (COVID-19 Genomics UK (COG-UK), COVID-19 Genomics UK Consortium., 2021). This study focuses on samples from HCWs and patients (including in-patients and those admitted to the ED), representing variants circulating in the hospital at the time. At times of high prevalence, when throughput was limited, samples were selected for sequencing based on the COG-UK surveillance sampling strategy. This strategy was employed by NHS Trusts across the COG-UK network to ensure that over 50% of local sequencing capacity was utilized towards national surveillance, ensuring that data represented a random representative subset of circulating variants. When capacity to sequence all available samples was not available, samples were selected randomly from those available each day up to at least 50% of local capacity. In addition, further samples were selected in a non-random way if they represented targeted sequencing priorities for national research studies, such as HCWs for the SARS-CoV-2 Immunity & Reinfection EvaluatioN (SIREN) study (https://snapsurvey.phe.org.uk/siren/). For the most part, local capacity remained high at PHU throughout this period, with 2,625 of 4,073 (64.4%) positive COVID-19 cases detected within QAH submitted for WGS.

### 2.4 Sample exclusion

**Figure 1:**
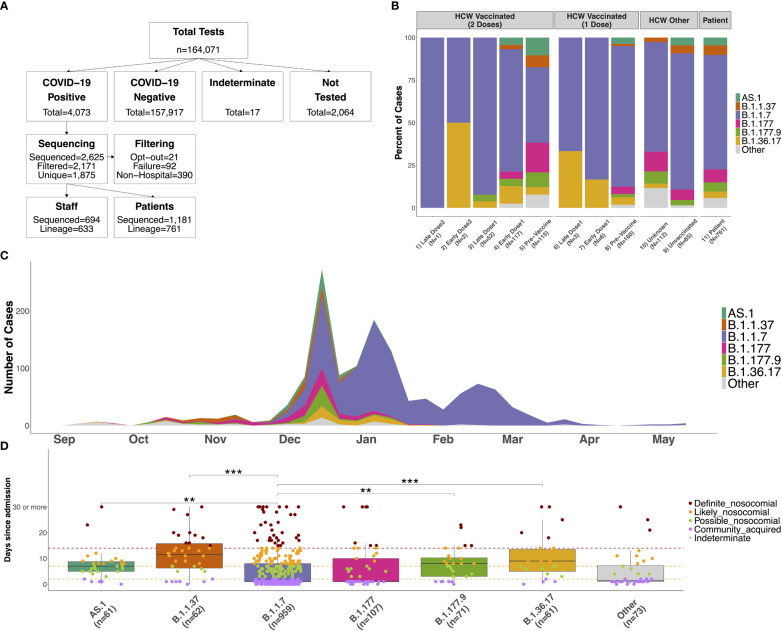
**(A)** Flow chart showing the number of COVID-19 tests performed between September 2020 and May 2021 at QAH, and the number of participants in the study. Positive samples were selected at random when it was not possible to process all samples. Those sequenced were removed when participants indicated their desire to opt-out from the study, due to failure of the sequencing (with no material available for repeating), or when the sample was from neither an in-patient nor HCW (these samples were included as community samples in downstream analyses). Only the first positive case was selected for individuals with multiple tests, and case numbers where a lineage could be determined using Pangolin are shown. **(B)** Distribution of lineages identified amongst SARS-CoV-2 samples for 1) HCWs with 2 doses testing positive 15 days or longer from the second vaccine dose (Late Dose2), 2) HCWs with 2 doses testing positive 14 days or fewer from the second vaccine dose (Early Dose2), 3) HCWs with 2 doses testing positive 15 days or longer from the first vaccine dose (Late Dose1), 4) HCWs with 2 doses testing positive 14 days or fewer from the first vaccine dose (Early Dose1), 5) HCWs with 2 doses testing positive prior to vaccination, 6) HCWs with 1 dose testing positive 15 days or longer from the first vaccine dose (Late Dose1), 7) HCWs with 1 dose testing positive 14 days or fewer from the first vaccine dose (Early Dose1), 8) HCWs with 1 dose testing positive prior to vaccination, 9) non-vaccinated HCWs, 10) HCWs with an unknown vaccination status (assumed non-vaccinated), or 11) patients within the hospital (primarily unvaccinated at this time). **(C)** Distribution of distinct lineages identified on a weekly basis at QAH. Distinct lineages are identified based on the Pangolin tool. Lineages with fewer than 5 cases are combined into a single class (‘Other’). Case numbers are here based on sequencing results only, so represent a subset of the total cases observed at QAH over this time. **(D)** Distribution of the number of days between admission and first testing positive for COVID-19 for patients within the major lineages identified amongst cases within the hospital. Nosocomial transmission is classified as being ‘definite’ if a positive test occurs greater than 14 days after admission (red), ‘likely’ if occurring greater than 7 days after admission (orange), ‘possible’ if occurring greater than 2 days after admission (green), and community-acquired otherwise (purple). Significant differences between groups are highlighted based on the results of a Wilcoxon rank sum test (*** <= 0.001; ** <= 0.01; * <= 0.05).

HCWs and in-patient samples were prioritized for downstream analyses, with other sample groups (e.g. out-patients and samples from non-hospital settings) classed amongst local community cases. Individuals who had indicated their preference to be excluded from the study through retrospective opt-out consent were removed, as were cases where the sequencing failed, with no material available for a successful repeat. Only the earliest positive case was taken forward for phylogenetic analysis for samples collected from the same individual. The final data set consisted of data from 694 HCWs and 1,181 patients (**Figure 1A**).

### 2.5 Whole genome sequencing

Sequencing was conducted following the ARTIC nCoV-2019 sequencing protocol V.3 (LoCost) (Quick, 2020). In brief, RNA was reverse transcribed and then amplified with amplicon PCR using the ARTIC nCoV-2019 V3 primer panel (Integrated DNA Technologies, Iowa, USA), which consists of 98 primer pairs tiling the full length of the ~30Kb SARS-CoV-2 genome. Split primer pools were used to prevent over-amplification of overlapping amplicon regions during the PCR. In addition, a negative nuclease-free water (NFW) control and a positive synthetic SARS-CoV-2 RNA control (Twist Bioscience, San Francisco, CA, USA) were added to each plate, which were used as quality control measures to monitor PCR success and contamination. Representative samples from each plate and positive and negative controls were quantified using the Qubit DNA Assay Kit in a Qubit 2.0 Fluorometer (Life Technologies, California, USA). Sequencing libraries were prepared for Nanopore sequencing using the Oxford Nanopore Technologies (ONT) LSK-109 Ligation Sequencing Kit and Nanopore EXP-NBD196 Native Barcoding Expansion 96 kit (ONT, Oxford, UK). Libraries were sequenced on R9.4.1 flow cells on a GridION X5 platform (ONT, Oxford, UK) for 24-36 hours (depending on library sample number) to achieve a final coverage of ~100,000 reads per sample. Raw reads were demultiplexed using the Guppy 3.2.10 toolkit integrated within the MINKnow software. The ARTIC fieldbioinformatics toolkit V1.2.1 (https://github.com/artic-network/artic-ncov2019) was used to process the resulting data. Reads were mapped against the SARS-CoV-2 reference genome (Wuhan-Hu-1, GenBank, MN908947.3) using MiniMap2 (v2.17-r941) (Li, 2018) and variants were identified using Nanopolish (v0.13.2; https://github.com/jts/nanopolish). Sequencing performance was monitored in real-time using the RAMPART (V1.0.6) software package (Mapleson et al., 2015). Variant assignment for resulting consensus sequences was conducted using Pangolin (https://github.com/cov-lineages/pangolin) with PANGOLearn version 2021-10-18. Samples with PCR contamination in the NFW negative control (defined by 200 reads or more mapping to the SARS-CoV-2 genome) were repeated.

### 2.6 Genomic data analysis

Multiple sequence alignments were generated using MAFFT (v7.310) (Katoh et al., 2002) for all HCWs and patients from QAH, along with cases from the latest COG-UK data on CLIMB (2021-02-18) from the Hampshire region as community cases. Iqtree (v2.1.2) (Nguyen et al., 2015) was used to generate phylogenetic trees using default parameters rooted on the reference genome (Wuhan-Hu-1, GenBank, MN908947.3) as an outgroup. Unless stated otherwise, all downstream data analyses were conducted using the R (v4.1.2) statistical programming language (R Core Team, 2021). The *ape* (v5.6-1) package was used to load the phylogenetic trees in R (Paradis and Schliep, 2019), which were plotted using *ggtree* (v3.2.1) (Yu, 2020). Clustering of cases was identified using the *transcluster* (v0.1.0) package in R (Stimson, 2018), with serial transmission interval (β) set to 5 days and the viral mutation rate (λ) set to 2 mutations per month based on previous estimates (Stimson et al., 2019; Meredith et al., 2020). Samples within 2 inferred transmission events (T) were clustered. Further analysis of transmission dynamics was conducted using the *a2bcovid* (v0.1.0) package in R (Illingworth et al., 2020). Statistical comparisons between count data were conducted based on the Chi-Squared test, whilst comparisons between numeric distributions were conducted using the non-parametric Wilcoxon rank-sum test. Figures were plotted using the *ggplot2* (v3.3.5) (Wickham., 2016) *ggpubr* (v0.4.0) (Kassambara, 2020) and *cowplot* (v1.1.1.) (Wilke, 2020) packages in R.

### 2.7 Epidemiological data analysis

Information collected for patient cases included: ward location, date of admission to hospital, date of testing positive and date of symptom onset. These data were collected from the Local Laboratory Information Systems (LIMS) using COGNOS for interrogation to identify all positive samples. Additional data on patient outcomes (death within 30 days of infection, intubation of the patient, and ICU admission of the patient) were collected were possible. Date of testing positive and shift location information were collected for HCWs, both as part of the QAH HCW screening program and manually from the APEX Pathology LIMS. These data were linked to SARS-CoV-2 genome sequence data using the COG-UK sequencing codes and locally assigned sample source IDs.

### 2.8 Definitions

We used clinical standards for defining the likelihood of nosocomial infection amongst patient samples based on the time from admission to testing positive (Huff and Singh, 2020): Community-acquired patient infections were those that tested positive 0-2 days after hospital admission; Possible nosocomial cases were those that tested positive 3-7 days after admission to the hospital; Likely nosocomial cases were those that tested positive 8-14 days after admission to the hospital; Definite nosocomial cases were those that tested positive 15 days or more after hospital admission. We defined a ‘transmission cluster’ is a group of SARS-CoV-2 cases with phylogenetic evidence of a potentially shared infection chain, and in particular focused primarily on those containing five or more individuals. Note that this may or may not include cases defined as nosocomial, as some may be part of shared community-based transmission chains.

### 2.9 Data availability

The consensus SARS-CoV-2 genomes and human-filtered sequencing data for COG-UK samples are routinely deposited in the European Nucleotide Archive (ENA) at EMBL-EBI under accession PRJEB37886. In addition, high-quality consensus genome files with coverage greater than 90% are routinely deposited to the Global Initiative for Sharing of All Influenza Data (GISAID) database. Accession numbers for anonymized samples featured in this study are available in **Supplementary Table 1**.

## 3 Results

### 3.1 Vaccination led to a reduced prevalence of COVID-19

Of the 6,810 HCWs at QAH, 5,964 (87.6%) had received at least one dose of the Pfizer/BioNTech BNT162b2 vaccine by the end of May 2021, with 4,203 (61.7%) having received two doses (**Table 1**). The prevalence of SARS-CoV-2 infection amongst HCWs was 10.0% in those that were unvaccinated, 2.4% within 14 days of the first dose and 1.3% 14+ days after the first dose. In this time-period, there were only two cases of COVID-19 within 14 days of the second dose and only one case of COVID-19 14+ days after the second dose in HCWs that had received two doses.

**Table 1:** Number of vaccinated and unvaccinated staff testing positive for COVID-19 within fewer than 14 days (early) or after 14 or more days (late) of their first or second vaccine dose *via* PCR testing for COVID-19.

	Unvaccinated	Vaccinated	+ve post-vaccine	+ve early (dose 1)	+ve late (dose 1)	+ve early (dose 2)	+ve late (dose 2)
**HCWs**	846	5,964 (1 Dose = 1,761) (2 Doses = 4,203)					
**Positive** **(All)**	85 (10.0%)	658 (11.0%)	223 (3.7%)	145 (2.4%)	78 (1.3%)	2 (0.0%)	1 (0.0%)
**Positive** **(2 doses)**		437 (7.3%)	211 (3.5%)	137 (2.3%)	74 (1.2%)	2 (0.0%)	1 (0.0%)
**Positive** **(1 dose)**		221 (3.7%)	12 (0.2%)	8 (0.1%)	4 (0.1%)		

### 3.2 The distribution of circulating SARS-CoV-2 variants in QAH changed as the Alpha variant prevailed

Between September 2020 and May 2021, a total number of 4,073 positive COVID-19 cases were detected within QAH (**Figure 1A**). Of these, 2,625 (64.4%) were selected for whole genome viral sequencing and filtered (see Materials and Methods) to produce a data set for 1,875 individuals. A total of 694 staff and 1,181 patient SARS-CoV-2 sequences were included in variant classification and transmission dynamics analyses (**Figure 1A**).

During this period, the six variants most commonly identified in positive COVID-19 samples at QAH were B.1.36.17, B.1.177, B.1.177.9, AS.1, B.1.1.37 and B.1.1.7 (**Figure 1C**). Before December 2020, cases within QAH were heterogeneous, with B.1.177 and B.1.1.37 in particular, each accounting for 16.4% of all cases. However, during December, the introduction of the B.1.1.7 Alpha variant into the community and the hospital resulted in a substantial increase in case numbers, reaching a peak in the first week of January 2021. During this initial rapid increase in cases, a significant increase in cases was also seen for other variants, including a 2.8-fold increase in cases of B.1.177, a 1.5-fold increase in cases of B.1.1.37, a 4-fold increase in B.1.177.9, and rapid expansion of previously rare variants AS.1 and B.1.36.17 (**Supplementary Figure 3**). However, B.1.1.7 soon became the dominant variant in QAH, with 100% of all sequenced samples showing this variant by February 2021 (**Figure 1C**; **Supplementary Figure 3**).

### 3.3 HCW COVID-19 cases represented all variants circulating in the hospital

The distribution of circulating variants was largely similar between patients (unknown vaccination status, but largely non-vaccinated at this time) and non-vaccinated HCWs (**Figure 1B**; χ^2^ = 6.81, df = 6, p = 0.339). A significant difference was detected between patients and HCWs with unknown vaccination status (**Figure 1B**; χ^2^ = 14.77, df = 6, p = 0.022), which was primarily a result of no cases of AS.1 amongst these individuals and an increase in multiple less-abundant variants (“Other”). A significant difference was also detected between patients and vaccinated HCWs who tested positive prior to their vaccine (**Figure 1B**; χ^2^ = 54.188, df = 12, p < 0.001). This difference is primarily the result of significant changes in the proportion of the B.1.1.7 variant over this time period. The vast majority of such cases, where a HCW tested positive prior to vaccination, will have occurred early in wave 2 prior to the vaccine program starting on 10th December 2020. Thus, pre-vaccination HCW cases contained very few cases of B.1.1.7 due to it not having emerged yet in most cases, leading to a higher proportion of B.1.1.7 cases identified in patient samples than pre-vaccination cases. Indeed, no difference was seen when removing this variant from the analysis (χ^2^ = 11.28, df = 10, p = 0.336). Since these HCWs were positive prior to beginning their vaccination, these similarly represent the background of infections amongst non-vaccinated staff. Therefore, infections in HCWs were largely representative of the variants circulating within the hospital at the time of testing positive, suggesting that there is no enrichment for any specific variants (which may be suggestive of HCW-specific outbreaks) amongst HCWs.

Only 15 HCWs tested positive after receiving their most recent vaccine dose during the study period (**Table 1**; **Figure 1B**). Given the later time point for these cases, the variants were primarily B.1.1.7, with several cases of B.1.36.17, which also saw some cases in January 2021. Double-vaccinated HCWs testing positive after the first dose showed differences in circulating variants compared to patients (χ^2^ = 31.55, df = 12, p = 0.002) due to a reduction in the presence of variants that decreased in their prevalence from January 2021. Importantly, whilst case rates were reduced amongst vaccinated HCWs, all circulating variants in QAH were identified amongst this group. Whilst increased proportions of B.1.1.7 were identified post-vaccination, these changes were likely the result of cases post-vaccination being primarily later in the timeline, when B.1.1.7 accounted for almost 100% of all cases. Indeed, B.1.1.7 was also predominant amongst patients by January 2021 (**Figure 1C**; **Supplementary Figure 4**). There is, therefore, no sign of significant vaccine escape for any of the variants observed in QAH at this time.

### 3.4 Nosocomial spread amongst circulating variants

The number of days from admission to receiving a positive test is typically used as an epidemiological definition of nosocomial infection (Huff and Singh, 2020). Comparing this metric amongst the circulating variants for patient COVID-19 cases highlighted that some variants may have contained more nosocomial cases than others. In particular, cases belonging to the B.1.1.37 and B.1.36.17 variants had a median number of 11.5 and 9 days since admission respectively amongst all cases (**Figure 1D**). This longer period from admission to infection suggests significant nosocomial spread amongst this group. Cases belonging to the AS.1 and B.1.177.9 variants had lower medians of 7 and 8 days post-admission respectively, whilst the variants associated with increased community prevalence, B.1.1.7 and B.1.177, each showed lower medians still of only 1-day post-admission (**Figure 1D**). A clear difference was seen between B.1.1.7 and all other groups except B.1.177, with significant differences detected between the days post-admission compared to AS.1 (p = 0.009), B.1.177.9 (p = 0.009), B.1.36.17 (p = 0.001) and B.1.1.37 (p < 0.001).

Based on epidemiological definitions of nosocomial infection and the time of infection post-admission (Huff and Singh, 2020), nosocomial cases were identified amongst all variants (**Table 2**). B.1.36.17 showed the greatest proportion of nosocomial cases in total (79.3% possible/likely/definite; 51.7% likely/definite), with few community-acquired cases identified (13.8%). Similarly, high levels of nosocomial cases were identified for B.1.1.37 (72.1% possible/likely/definite; 58.2% likely/definite) and AS.1 (71.4% possible/likely/definite; 37.1% likely/definite). In contrast, a smaller proportion of B.1.1.7 infections were nosocomial (30.7% possible/likely/definite; 18.6% likely/definite), with 37.8% identified as community-acquired, indicating that B.1.1.7 was primarily driven by community spread. Prevalence of community spread was also seen for B.1.177, with fewer nosocomial cases (37.9% possible/likely/definite; 24.2% likely/definite) and a higher proportion of community-acquired cases (37.9%).

**Table 2:** Number of patients within each nosocomial group within the primary viral lineages circulating.

	Total patients	Definite nosocomial	Likely nosocomial	Possible nosocomial	Community- acquired	Indeterminate	Total nosocomial
**B.1.1.7**	511	33 (6.5%)	62 (12.1%)	62 (12.1%)	193 (37.8%)	161 (31.5%)	157 (30.7%)
**B.1.177**	58	7 (12.1%)	7 (12.1%)	8 (13.8%)	27 (46.6%)	9 (15.5%)	22 (37.9%)
**B.1.1.37**	43	11 (25.6%)	14 (32.6%)	6 (14.0%)	7 (16.3%)	5 (11.6%)	31 (72.1%)
**B.1.177.9**	41	4 (9.8%)	11 (26.8%)	7 (17.1%)	6 (14.6%)	13 (31.7%)	22 (53.7%)
**AS.1**	35	2 (5.7%)	11 (31.4%)	12 (34.3%)	5 (14.3%)	5 (14.3%)	25 (71.4%)
**B.1.36.17**	29	5 (17.2%)	10 (34.5%)	8 (27.6%)	4 (13.8%)	2 (6.9%)	23 (79.3%)
**Other**	44	3 (6.8%)	6 (13.6%)	5 (11.4%)	22 (50.0%)	8 (18.2%)	14 (31.8%)
**None**	420	19 (4.5%)	32 (7.6%)	44 (10.5%)	139 (33.1%)	186 (44.3%)	95 (22.6%)

All lineages with fewer than 5 total cases (including HCWs) were combined into a single group (‘Other’). Community-acquired = 0-2 days post-admission; Possible-nosocomial = 3-7 days post-admission; Likely-nosocomial = 8-14 days post-admission; Definite-nosocomial = 15+ days post-admission.

### 3.5 Identification of shared transmission chains using viral genomics

**Figure 2:**
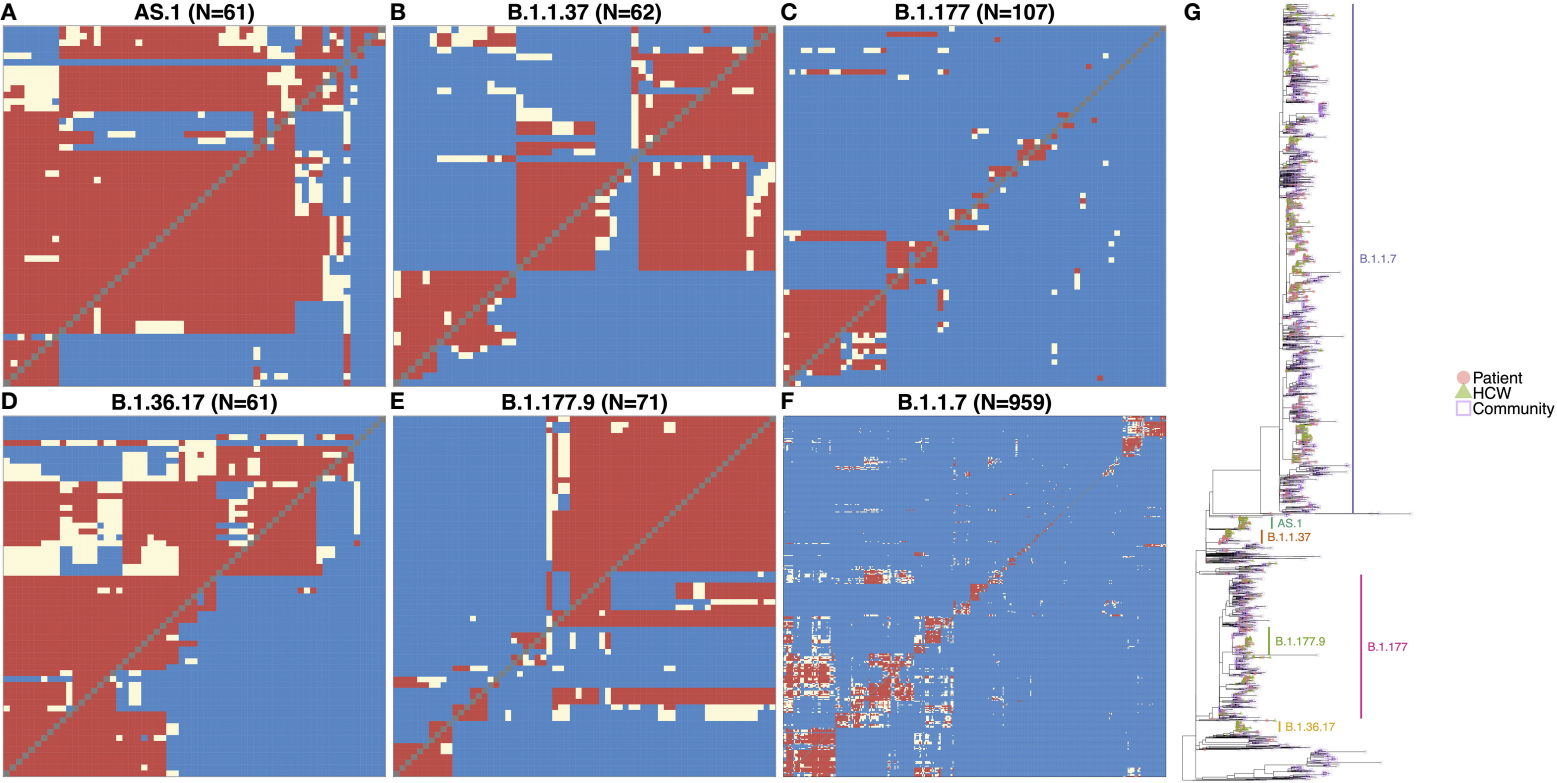
**(A–F)** Transmission dynamic plots based on analysis using the a2bcovid package in R for individuals with one of the 6 major lineages circulating within the hospital during the period of the study. Pairwise likelihood of direct transmission between cases is encoded as ‘Consistent’ (red) for likely cases of person-to-person transmission, ‘Borderline’ (cream) for potential cases of person-to-person transmission, and ‘Unlikely’ (blue) for unlikely cases of person-to-person transmission. Healthcare workers are highlighted in red. **(G)** Phylogenetic tree of SARS-CoV-2 genomes from staff and patients identified at QAH through the study period between September 2020 and May 2021, along with community cases from the city of Portsmouth, and the county of Hampshire in the UK. The tree is rooted on the early SARS-CoV-2 genome MN908947.3, with distance on the x-axis corresponding to evolutionary distance from this progenitor. Similar genomes are clustered into lineages, with the most significant lineages circulating within the hospital at this time highlighted. Node tips are colored based on whether they represent patients (filled red circle), healthcare workers (filled green triangles), community cases from the city of Portsmouth (empty blue squares), or community cases from the county of Hampshire (empty purple diamonds). Community cases are represented by open symbols to make QAH samples easier to identify.

SARS-CoV-2 genome variants can differ by many mutations, and identifying the specific variant alone is not suitable for determining directly linked cases and nosocomial transmission clusters within the hospital. However, phylogenetic cluster analysis of the viral whole-genome sequences, combined with information on likely infection dates and shared locations, enabled the identification of within-variant clusters amongst HCWs and patients at QAH, resulting in the elucidation of possible linked transmission chains and nosocomial clusters (**Figures 2A–F**). Of the six variants most commonly detected within QAH in this period, within-variant pairwise comparisons indicated that the proportion of pairwise cases consistent with direct transmission was high, suggestive of a single major outbreak for AS.1 (**Figure 2A**) and B.1.36.17 (**Figure 2D**); moderate, suggestive of multiple distinct outbreaks for B.1.1.37 (**Figure 2B**) and B.1.177.9 (**Figure 2E**); and low, suggestive of community spread with multiple potential local outbreaks for B.1.177 (**Figure 2C**) and B.1.1.7 (**Figure 2F**). Plotting the phylogenetic tree in the context of other cases from the local community and surrounding region further highlights that whilst AS.1, B.1.36.17, B.1.1.37 and B.1.177.9 were enriched amongst cases from QAH, suggesting the likelihood of nosocomial spread, cases of B.1.1.7 and B.1.177 from QAH were seen in amongst a significant proportion of community cases, suggesting that nosocomial spread was not the primary driver of infection for cases amongst these lineages (**Figure 2G**). Whilst many of these nodes contained mostly community cases or events in which outbreaks in the community appeared to be brought into the hospital by HCWs and patients, some appeared to indicate outbreaks within QAH (**Figure 2G**).

### 3.6 Transmission dynamics for AS.1

**Figure 3:**
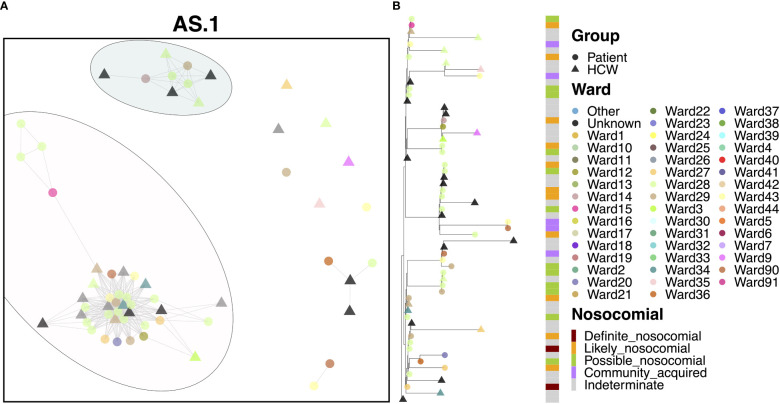
**(A)** Cluster plot for the AS.1 lineage based on analysis using the transcluster package in R. Individual samples are represented by circles (patients) or triangles (HCWs), with the color representing the anonymized ward on which the patient was tested. Samples are connected in the graph if they show evidence of being within 2 transmissions or fewer from one another to highlight linked infections. **(B)** The sub-phylogeny for the specific lineage is also shown, colored by anonymized ward, with likelihood of nosocomial infection (based on time since admission) indicated in the bar alongside.

Most cases of variant AS.1 were identified amongst two distinct transmission clusters (**Figure 3A**). The larger of the two groups consisted of 25 patients and 14 HCWs, with the earliest case originating from a patient on Ward 15 in November. Interestingly, this case was itself designated as ‘likely’ nosocomial, indicating that there may be earlier cases in this transmission chain not accounted for in our HCW and patient data. This difference may indicate initial infection from an asymptomatic carrier or from a visitor to the hospital. Several infections occurred amongst staff and patients, with a large rapid expansion of cases consistent with an outbreak on Ward 28 (15 patients and at least one HCW known to be on this ward), which accounted for the majority of cases. Patients in this group were primarily ‘possible’ or ‘likely’ nosocomial cases, although no ‘definite’ cases on the ward for longer than 2 weeks were identified (**Figure 3B**). Whilst there was significant spread on this ward, the variability amongst cases indicates that this is unlikely the result of a super-spreader event. While the initial outbreak was spread to other wards, few of these events resulted in similar outbreaks. Similarly, whilst infection of HCWs was identified as a result of this outbreak, these HCWS did not themselves act as vectors to seed further outbreaks in other areas of the hospital. Interestingly, several cases were detected downstream in the Emergency Department (ED) through routine swabbing of patients, classed as community transmission by the epidemiological definitions. The smaller of the two clusters consisted of four patients and five HCWs and appears to be an off-shoot of the major cluster, again seen primarily on Ward 28.

### 3.7 Transmission dynamics for B.1.1.37

**Figure 4:**
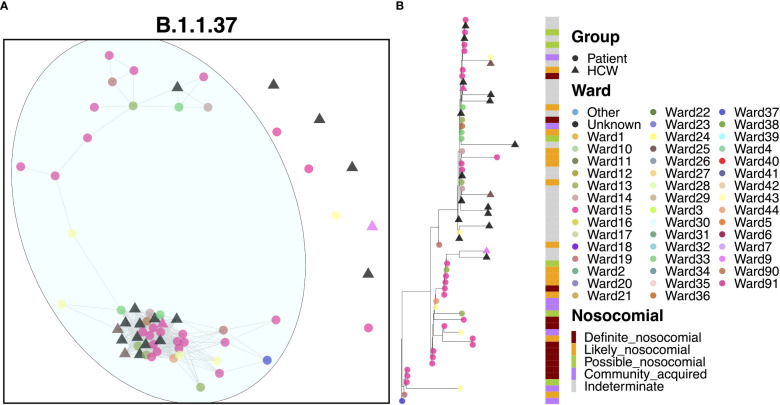
**(A)** Cluster plot for the B.1.1.37 lineage based on analysis using the transcluster package in R. Individual samples are represented by circles (patients) or triangles (HCWs), with the color representing the anonymized ward on which the patient was tested. Samples are connected in the graph if they show evidence of being within 2 transmissions or fewer from one another to highlight linked infections. **(B)** The sub-phylogeny for the specific lineage is also shown, colored by anonymized ward, with likelihood of nosocomial infection (based on time since admission) indicated in the bar alongside.

Apart from a handful of cases, the vast majority of cases (39 patients and 14 HCWs) appeared to be linked to a single large outbreak (**Figure 4A**). At least 22 of these cases were identified on Ward 15, and 59.0% of patients were ‘definite’ or ‘likely’ nosocomial cases (**Figure 4B**). The earliest case in this cluster was a community-acquired case on Ward 37, but one week later, an outbreak of nosocomial cases was detected on Ward 15, also identified for early cases of AS.1. This outbreak continued for nearly two months although interestingly, no HCWs were infected within the outbreak until much later in December, suggesting that PPE measures worked well to prevent onward spread to HCWs. Onward spread to other areas within the hospital occurred, but further outbreaks were not detected on other wards. Interestingly, there were six cases where the time to infection was suggestive of community spread, including cases detected in the ED. However, given their positions within the phylogenies, it is likely that these represent downstream infections and may indeed be nosocomial cases with a short incubation time. A distinct pattern within this cluster was that patients appeared to pass infections on to each other and HCWs, whereas HCWs did not transmit infections to patients.

### 3.8 Transmission dynamics for B.1.177

**Figure 5:**
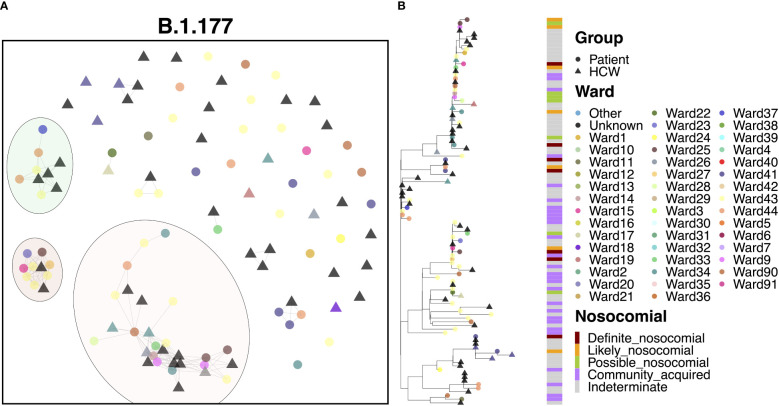
**(A)** Cluster plot for the B.1.177 lineage based on analysis using the transcluster package in R. Individual samples are represented by circles (patients) or triangles (HCWs), with the color representing the anonymized ward on which the patient was tested. Samples are connected in the graph if they show evidence of being within 2 transmissions or fewer from one another to highlight linked infections. **(B)** The sub-phylogeny for the specific lineage is also shown, colored by anonymised ward, with likelihood of nosocomial infection (based on time since admission) indicated in the bar alongside.

The B.1.177 variant was a highly abundant SARS-CoV-2 lineage that showed significant expansion in the UK from Europe in the summer of 2020 in response to the re-opening of borders for travel. In line with the increased community prevalence of this variant, cases of B.1.177 in QAH appeared to be primarily defined by community spread, with only 6.5% ‘definite’ nosocomial, 5.5% ‘likely’ nosocomial and 7.5% ‘possible’ nosocomial cases. Whilst most cases appeared to be independent introductions from the community, three clusters were identified, accounting for 27.1%, 9.3% and 8.4% of cases, respectively (**Figure 5A**). The smaller cluster consisted of four HCWs and five patients who tested positive within one day of admission, collected over the course of two months. Given the short time of positive testing following admission, along with long times between successive positive cases within this cluster (median 9 days), and the fact that patient cases in this cluster were primarily identified in the ED, it is likely that these cases were part of a transmission chain external to the hospital itself, and not representative of nosocomial spread. The mid-sized cluster also appeared to represent independent introductions through the ED of cases of a transmission chain from the community. However, there were three later cases of infection in patients admitted for 14 days or more (**Figure 5B**). The point of infection is not immediately clear, with all three being on distinct wards and occurring over 12 days, with only a single HCW infection identified within this time. However, this would indicate no clear systematic point of infection and may instead represent additional independent infections from a transmission chain active in the community through visitors to the hospital, including those visiting patients and attending outpatient appointments. This explanation is supported by multiple additional community cases in the ED identified between these nosocomial-classed cases. There was a similar pattern for the largest cluster, with 29 cases identified over a period of 1.5 months, with 13.8% ‘likely’ nosocomial and 17.2% ‘possible’ nosocomial. However, most cases (75.9%) occurred within 12 days, including multiple cases on shared wards. In particular, three of the earliest cases (one patient and two HCWs) occurred on a single ward, with additional cases later identified on this and neighboring wards. Given the timing of the cases (all three occurred over two days), it is difficult to identify whether onward spread was through transmission from patients or HCWs in this case.

### 3.9 Transmission dynamics for B.1.36.17

**Figure 6:**
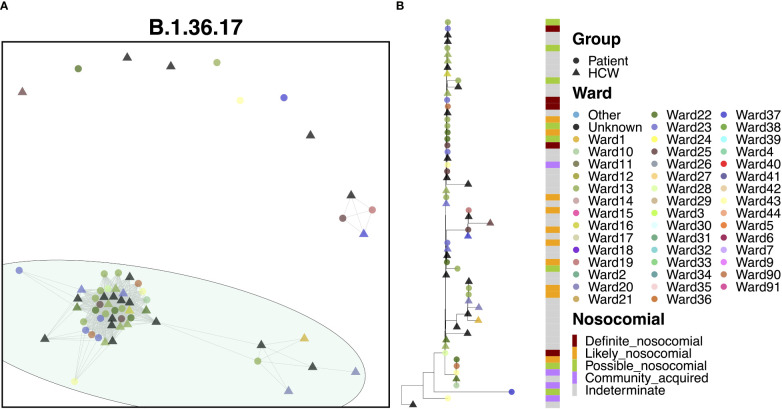
**(A)** Cluster plot for the B.1.36.17 lineage based on analysis using the transcluster package in R. Individual samples are represented by circles (patients) or triangles (HCWs), with the color representing the anonymized ward on which the patient was tested. Samples are connected in the graph if they show evidence of being within 2 transmissions or fewer from one another to highlight linked infections. **(B)** The sub-phylogeny for the specific lineage is also shown, colored by anonymized ward, with likelihood of nosocomial infection (based on time since admission) indicated in the bar alongside.

80.3% of cases of B.1.36.17 belonged to a single transmission cluster (**Figure 6A**), including 23 patients and 26 HCWs, with the earliest cases within this cluster belonging to two patients testing positive at admission to a medical ward (the earliest on Ward 25, the second three days later on Ward 23). Interestingly, the earliest case of B.1.36.17 as a whole within the hospital was from an HCW (**Figure 6B**), but this case was seen nearly two months prior to establishment of the major transmission cluster. Early cases were identified on Ward 23, which spread onto neighboring Ward 22 and another nearby ward within the same building, Ward 13. This cluster displayed a rapid expansion of almost identical cases (17 patients and 17 HCWs), largely centered around Wards 13 and 23, indicative of a ‘super-spreader’ event. Given the timings of infections, it is likely that the second earliest patient was admitted to Ward 23 carrying their infection from the community (possibly asymptomatically) and infected many staff and patients on the ward before their infection was identified through PCR testing. However, given the long incubation time of the virus, the date of testing positive may not be an accurate representation of infection time and so it is also possible that the initial spread came from elsewhere.

### 3.10 Transmission dynamics for B.1.177.9

**Figure 7:**
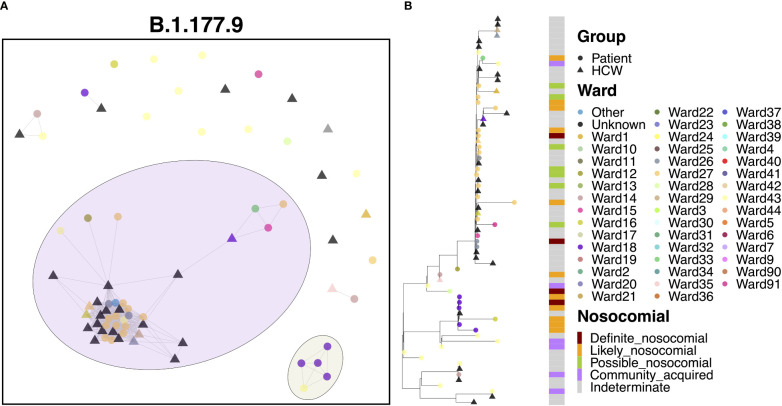
**(A)** Cluster plot for the B.1.177.9 lineage based on analysis using the transcluster package in R. Individual samples are represented by circles (patients) or triangles (HCWs), with the color representing the anonymized ward on which the patient was tested. Samples are connected in the graph if they show evidence of being within 2 transmissions or fewer from one another to highlight linked infections. **(B)** The sub-phylogeny for the specific lineage is also shown, colored by anonymized ward, with likelihood of nosocomial infection (based on time since admission) indicated in the bar alongside.

Cases of B.1.177.9 fell primarily into two main clusters, with 42 (59.2%) cases in the larger of the clusters and 5 (7.0%) cases in the smaller transmission cluster (**Figure 7A**). The smaller of the two clusters was the earlier of the two, with 5 patient cases seen in October 2020. The earliest of these cases appeared to have been identified in the ED, with further spread occurring two weeks later onto Ward 18, with all four patients showing ‘likely’ or ‘definite’ nosocomial infection (**Figure 7B**). Interestingly, this variant was also enriched amongst the local community and spread throughout October (**Figure 2G**) based on targeted sequencing of students from the University of Portsmouth (data not shown). Therefore, this is likely an introduction of a community-acquired infection through the ED, resulting in a small outbreak on Ward 18. On the other hand, the larger of the two clusters began later in December, consisting of 21 patients and 21 HCWs, and may represent an independent introduction to the hospital. Indeed, the first eight cases in this cluster consisted of six patients, all of whom had an ‘indeterminate’ nosocomial status, including at least one case from the ED. This variant resulted in an outbreak seemingly enriched on Ward 27 (14 cases), which later spread to neighboring Ward 26 (3 cases).

### 3.11 Transmission dynamics for B.1.1.7

**Figure 8:**
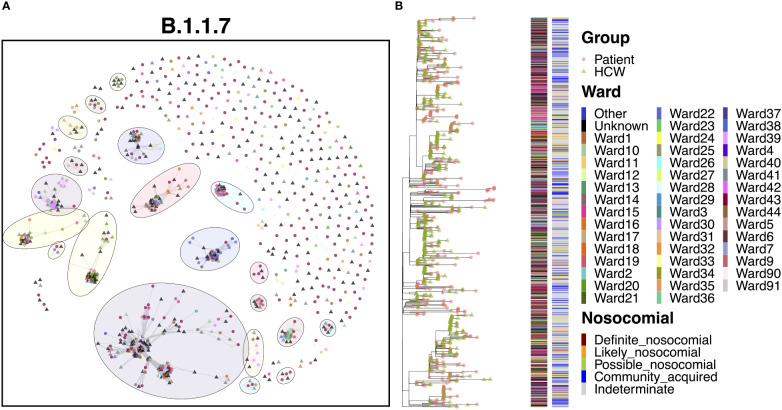
**(A)** Cluster plot for the B.1.1.7 lineage based on analysis using the transcluster package in R. Individual samples are represented by circles (patients) or triangles (HCWs), with the color representing the anonymized ward on which the patient was tested. Samples are connected in the graph if they show evidence of being within 2 transmissions or fewer from one another to highlight linked infections. **(B)** The sub-phylogeny for the specific lineage is also shown, colored by anonymized ward, with likelihood of nosocomial infection (based on time since admission) indicated in the bar alongside. .

The B.1.1.7 variant represents the Alpha variant of concern, which came to prominence over the Christmas period in 2020. Given the significant increase in cases throughout this study due to the spread of Alpha, it is no surprise that this variant represented a marked increase in cases compared to the previous variants (**Figure 8A**). As with B.1.177, B.1.1.7 was characterized by a significant level of community spread, with only 6.5% ‘definite’ and 12.1% ‘likely’ nosocomial cases (**Table 2**). However, there is evidence of shared infections from sequence comparisons of clustered cases, with 20 clusters of five or more cases (550 cases) and nine clusters of 10 or more cases (474 cases). Whilst many cases appeared to be independent introductions to the hospital from the community, 49.4% of cases were found in clusters of 10 or more cases. The largest cluster, consisting of 198 cases (20.6%), included 33 cases from the ED and 11 cases from COVID-19 high care wards (both consistent with the community spread seen with B.1.177), as well as groups of patients on several different wards, indicating nosocomial spread within the hospital. The earliest cases over the first month were almost all patient samples identified in the ED or COVID-19 high care wards (60.0%), with the first ward infection occurring two weeks after the initial introduction and the first HCW infection occurring a further three days later. Following these early cases, many cases occurred in a short period, with 115 cases (58.1%) occurring within a mean time difference of 0.2 days from one another over a period of only 26 days, suggestive of significant onward spread. However, it is interesting that only six of these cases were ‘definite’ and seven were ‘likely’ nosocomial based on admission date, with a large proportion (16.5%) still identified in the ED and high care wards. In addition, whilst spread was detected within the hospital, it was not confined to a single ward as seen in cases of super-spreader events for other variants (**Figure 8B**). Therefore, such clusters may represent multiple introductions of larger transmission chains prevalent in the community. Indeed, the ED accounted for a large proportion (23.4%) of all B.1.1.7 cases, whilst other cases were typically spread across a wide range of wards, highlighting multiple independent introductions to the hospital from the community across all clusters.

### 3.12 Severe outcomes are not increased in nosocomial cases

To understand whether nosocomial cases of SARS-CoV-2 infection were more likely to result in severe outcomes for patients, we looked at three measures of severity; death within 30 days of infection, intubation of the patient, and ICU admission of the patient. We were able to link outcome data to our filtered genomic data set for 625 out of 1,181 patients (including inpatients and patients testing positive in ED who may have subsequently become inpatients). Data from genomic cluster analyses were combined to identify ‘true’ nosocomial cases as those possible, likely and definite nosocomial cases that are part of a transmission cluster of 5 or more cases in PHUT. Other cases were classified as being likely community-acquired infections. 94 indeterminate cases were removed from the analysis, leaving a final data set of 531 patient cases.

The proportion of cases identified as nosocomial based on time from admission with genomic evidence of being nosocomial was 59.8%, 74.6% and 78.7% for possible, likely and definite nosocomial cases respectively. This indicates that many cases seen in patients between 3- and 7-days following admission may in fact be community acquired with a long incubation period. Conversely, 39.4% of cases identified as community-acquired showed genomic evidence of being part of a shared transmission cluster with others in the hospital. Thus, nosocomial rates identified based on both time from admission and genomic evidence may not fully represent the true rate of hospital-acquired transmission. However, whilst potentially underestimating the nosocomial incidence, cases with evidence from both approaches are likely to represent ‘true’ nosocomial cases.

With these caveats, we used the combined approach for defining nosocomial cases to explore effects of transmission dynamics with patient outcomes and severity. Initial Chi-squared comparisons of severity against ‘true’ nosocomial state identified significant effects of nosocomial status of the infection on death (χ^2^ = 8.40; df = 1; p = 0.004), intubation (χ^2^ = 9.04; df = 1; p = 0.003), and ICU admission (χ^2 =^ 4.22; df =1; p = 0.040). Interestingly, whilst death rates were higher amongst nosocomial cases (40.2%) compared to community cases (27.6%), the opposite was true for intubation and ICU admission, with higher rates seen amongst community cases (13.9% and 16.0% respectively) than amongst nosocomial cases (5.2% and 9.3% respectively).

However, given that many nosocomial outbreaks were linked to a small number of rapid expansions and often associated with infections on wards caring for elderly and vulnerable patients, the nosocomial group was skewed for older (mean age 78.9 ± 13.7) and more vulnerable patients compared to the community group (mean age 69.1 ± 16.8), with a significant difference seen for the two groups (t = 7.29; df = 470.04; p < 0.001). Similarly, the nosocomial group typically showed a longer length of stay (26.7 ± 20.9 days) compared to the community group (15.7 ± 19.0 days), with a significant difference seen for the two groups (t = 6.05; df = 371.21; p < 0.001). Interestingly, however, the correlation between the patient age and length of stay was low (ρ = 0.06; p = 0.203), indicating that the two were not generally directly related.

Logistic regression, with severity as the response variable and nosocomial status as the explanatory variables, but with age and length of stay included as covariates, identified significant associations with age, no association with length of stay, but importantly no association with nosocomial status for death (ß = 0.27; SE = 0.21; p = 0.204). Interestingly, the roughly 3-fold decrease in the rate of intubation seen in nosocomial cases (5.2%) compared to community cases (13.9%) remained highly significant even after correcting for patient age and length of stay (ß = -1.49; SE = 0.44; p < 0.001). Similarly, the roughly 2-fold decrease in the rate of ICU admission seen in nosocomial cases (9.3%) compared to community cases (16.0%) remained significant after correcting for patient age and length of stay (ß = -0.86; SE = 0.36; p = 0.018).

Whilst age and length of stay were higher within the nosocomial group, suggesting that nosocomial patients were enriched for more vulnerable patients, the average National Early Warning Score (NEWS2) at admission was significantly lower (t = -8.28; df = 475.75; p < 0.001) for nosocomial cases (2.38 ± 2.68) compared with community cases (4.58 ± 3.35). The NEWS2 score is a simple to calculate aggregate bedside scoring system, with higher scores being associated with increased risk of acute illness. In comparison, no difference was seen for the maximum NEWS2 score (t = -0.28; df = 384.07; p=0.779) between nosocomial cases (6.15 ± 3.23) and community cases (6.23 ± 3.02). This therefore suggests that nosocomial cases were generally less severe at admission but became as severe as community cases over time.

## 4 Discussion

The detailed genomic analyses conducted in this investigation revealed that there were six primary variants of SARS-CoV-2 circulating within QAH during the second wave of the COVID-19 pandemic. There were varying levels of nosocomial transmission within each of the variants, indicating that the spread of SARS-CoV-2 within the hospital was not always a direct result of transmission between patients and HCWs within the hospital itself but due to multiple independent introductions that occurred as a result of high community prevalence. For example, B.1.36.17, B.1.1.37, and AS.1 showed particularly high proportions of nosocomial infections, with 79.3%, 72.1% and 71.4%, respectively (**Table 2**), indicating nosocomial spread as the primary source of transmission. In contrast, the Alpha variant B.1.1.7 and the B.1.177 variant, both associated with significant community incidence, had comparatively low levels at only 30.7% and 37.9% respectively, suggesting that community transmission was far more prevalent in these cases. So, whilst suspected nosocomial transmission was seen for both variants, this was not the primary driver of viral transmission. However, whilst the time between admission and infection can indicate the likelihood of infection occurring within the hospital, this measure may not always be accurate when attempting to infer transmission dynamics. For instance, a ‘definite nosocomial’ case identified in a patient on the ward for 15 days may be isolated and not part of any ongoing transmission chains within the hospital if it was transmitted from the community by a hospital visitor or newly admitted patient. In contrast, a patient in the ED may become infected from an ongoing transmission chain within the hospital and yet be classified as ‘community-acquired’ based on this measure.

We have demonstrated that SARS-CoV-2 genomic surveillance and cluster analyses are powerful tools for understanding nosocomial outbreak transmission dynamics. Four large SARS-CoV-2 variants expanded at QAH during the peak of the second pandemic wave, each of which encompassed multiple wards and has evidence of being primarily driven by nosocomial spread. This observation is a notable finding, considering that outbreaks are currently managed geographically on a ward-by-ward basis, with limited understanding of how the transmission may occur between wards. In addition, we uncovered variant-specific transmission dynamics for each of the four large transmission clusters, which were characterized by factors such as uncontained community-introduced cases and potential ‘super-spreader’ patients.

The increased prevalence of the Alpha variant between December 2020 and January 2021 in the local community appears to have resulted in a significant expansion of many variants circulating within the hospital. Onward spread will have likely been compounded by the difficulty in maintaining infection control procedures and case isolation as infections rose. This difference was highlighted by the genomic analyses, which were able to identify those cases likely to be linked as part of a single transmission cluster from those that represent independent infections and introductions from the community. When linked to timing data between admission and testing positive, clustering cases based on WGS provides a powerful approach for determining true nosocomial transmission. Following the initial expansion of cases, the presence of the non-Alpha variants was significantly reduced, with almost all cases having disappeared by the end of January 2021.

Similarly, whilst there were clusters of linked cases likely to represent nosocomial spread for the Alpha variant, the majority of cases likely represent unique introductions from the community. However, it is worth noting that the Alpha variant has been shown to have a decreased incubation time compared to other variants (Blanquart et al., 2022), leading to nosocomial infections occurring within shorter timescales than seen for previous variants. Thus, the definitions used in this analysis may in fact under-estimate the true scale of nosocomial infections for Alpha cases, in particular for likely and definite cases. It is therefore possible that the proportion of nosocomial cases within Alpha clusters is higher than expected. However, it is interesting to note that super-spreader type events, characterized by rapid transmission within single wards in the hospital, were not prevalent. This further highlights the difficulties facing IPC teams in identifying true nosocomial infections, particularly when prevalence is high.

Improved infection control measures and rapid testing were introduced in the hospital in early 2021 (**Supplementary Figure 1**), which helped to address rising infection rates. In addition, HCWs at QAH were amongst the first recipients in the UK of the Pfizer-BioNTech (BNT162b2) vaccine. Both interventions coincided with a reduced prevalence of COVID-19 infection in the hospital, although it must be noted that case numbers were decreasing in this period regardless, in response to non-pharmacological interventions introduced by the UK Government. Nonetheless, HCWs that received at least one dose of the vaccine had a lower prevalence of COVID-19 infection than those not vaccinated (**Table 1**). Interestingly, despite the lower prevalence amongst vaccinated staff, the rate of HCWs shown to be part of a cluster of 5 or more cases within the hospital was only slightly lower in vaccinated (61.9%) and unvaccinated (64.1%) HCWs, indicating that the vaccine status of HCWs did not have a significant impact on their likelihood of being part of a transmission chain within the hospital (χ^2^ = 0.17, df = 1, p = 0.682). So, whilst vaccinated staff had a lower overall incidence of COVID-19 than non-vaccinated staff, those who tested positive were equally likely to be infected within any ongoing transmission chains. This is similarly highlighted because infections in vaccinated and unvaccinated staff appear to be largely represented by circulating variants (**Figure 1B**).

One of the most important questions to help understand and prevent nosocomial infections is how the virus was first introduced into the hospital. These analyses suggest that one of the primary entry points may have been *via* the ED and from patients testing positive shortly after admission, having brought the virus in from the community. This is particularly true for the B.1.177 and B.1.1.7 Alpha variants, which were characterized by increased community prevalence. Isolation of patients admitted to the ED with suspected COVID-19 infection and newly-admitted patients awaiting a PCR test result, along with the use of rapid tests such as antigen-based lateral flow devices and RT-LAMP assays, may therefore halt transmission chains for which these are the entry points. Whilst these measures were in place in hospitals such as QAH, it must also be noted that during this second wave of infections in the UK, many of these nosocomial outbreaks occurred when COVID-19 case numbers were at their peak, largely as a result of the unprecedented spread of the Alpha variant. The decreased incubation period of this variant (Blanquart et al., 2022) likely enabled it to spread more rapidly than control measures could have been put in place. Hospitals across the country were at full capacity during this time, and so sufficient isolation of patients may have been difficult to manage, if not impossible. In most of the large transmission clusters identified at QAH, it was rare for HCWs to transmit the infection to patients, suggesting that HCWs represent a low risk in terms of acting as an entry point for the virus into the hospital or as vectors for transmission between wards. This reduced risk is likely a reflection of the effectiveness of PPE, routine HCW testing within the hospital and general efforts made by staff to follow guidelines on physical distancing. Control measures are likely more effective at preventing transmission from HCWs than they are for patient transmission (Lindsey et al., 2022). Indeed, the high number of HCW samples present within this dataset (37.0%) results from regular asymptomatic screening of staff within the hospital.

To understand the role that nosocomial transmission of SARS-CoV-2 played in disease outcome, we classified patients into those with both epidemiological and genomic evidence of nosocomial transmission, and those where the combined evidence suggests that infections may have been community driven. This showed that over 40% of cases seen in patients within 3-7 days from admission show little evidence of shared infections within the hospital, suggesting that they may be community infections with a long incubation time misclassified as nosocomial infections. The same is true for over 20% of likely and definite nosocomial cases. In such cases, infection may have occurred within the hospital, but as a result of a novel introduction from the community. Given that HCW screening was performed regularly, such introductions may result from visitation from pre- or asymptomatic members of the public, highlighting regular testing of visitors to the hospital as a key area for minimizing infection transmissions. However, conversely nearly 40% of community-acquired infections showed evidence of being part of a shared infection chain with other cases within PHU. This indicates that some cases seen in patients up to 2-days following admission may in fact be hospital-acquired, albeit with a rapid incubation time. It is also possible that clusters seen within the hospital represent subsets of infections circulating within the community at large. Thus, fully resolving hospital-acquired and community-acquired infections remains a challenge for IPC teams. However, whilst potentially underestimating nosocomial incidence, cases with evidence from both approaches are likely to represent ‘true’ nosocomial cases.

Understanding the factors that influence the severity of COVID-19 in patients remains a key question. We used this estimate of ‘true’ nosocomial cases to observe any effects on severity of COVID-19 resulting from nosocomial infections. These data suggest that nosocomial infections are no more likely to result in mortality from COVID-19 than community-acquired infections after accounting for age and length of hospital stay. Instead, hospital-acquired COVID-19 infections are likely to preferentially affect older and more vulnerable individuals in hospital for longer periods of time, who are more at risk. Indeed, similar mortality rates between hospital and community acquired infections have been previously reported, with advanced age and frailty of the patients identified as biases likely to underlie this association (Ponsford et al., 2021). It was interesting to note that the NEWS2 score for the nosocomial cases was actually lower at admission than those within the community group. This may again link to the fact that these patients were often older patients in long term care, rather than patients admitted for acute illness. However, the maximum NEWS2 scores for both groups were almost identical, indicating that the results of COVID-19 related pulmonary pneumonitis on acute illness are common regardless of the mode of infection. Interestingly, both ICU admission and intubation were lower in cases identified as nosocomial as compared to community cases. These data and the factors most influencing severity of COVID-19 infections will be explored further in future studies.

A striking finding from our analyses, confirmed by other genomic investigations of SARS-CoV-2 transmission in various healthcare and social settings, is evidence for ‘super-spreaders’ and the severe impact of these individuals in outbreaks (Adam et al., 2020). This is a phenomenon whereby one individual transmits the infection to many other individuals, as seems to be the case for the B.1.36.17 outbreak at QAH. At Cambridge University Hospitals NHS Foundation Trust, up to 80% of nosocomial infections were caused by approximately 20% of patients during the first wave of the pandemic (Illingworth et al., 2021), which occurred between March and June 2020. Many factors may contribute to super-spreading events (SSEs) (Wong et al., 2015), and the impacts of these events are usually severe because they are not identified quickly enough to be reasonably contained. Future research into what makes a patient a super-spreader, such as high viral load (Avadhanula et al., 2021), will be pivotal for informing how potential super-spreaders can be quickly identified and isolated. PCR testing at this time in PHU was performed using the Hologic Panther Fusion system, which does not provide Ct scores to quantitatively assess viral load. This unfortunately prevented assessment of individuals within the B.1.36.17 cluster to understand whether a higher than normal viral load may have impacted on transmission.

All of the SSEs that have been described for SARS-CoV-2, including those in the present investigation, have been defined as SSEs retrospectively using either epidemiological or genomic data, weeks or months after the event occurred. Rapid turnaround WGS and application of tools that we have used in this investigation, such as A2B Covid, genomic clustering and phylogenetics, could enable outbreaks to be disentangled in precise detail within the week that they are initiated, which may enable precise control measures to be put in place soon enough to contain the outbreak quickly. Such rapid feedback from WGS has been trialed during the second wave of COVID-19 in the UK through the Hospital Onset COVID-19 Infection (HOCI) study, which shows the effectiveness of rapid genotyping of patient infections and identification of linked cases for impacting Infection Control Procedures (Stirrup et al. 2021; Stirrup et al., 2022). The limitations of this approach are that it requires easy access to WGS and specialist knowledge of bioinformatics, which may not be readily available within all hospital settings.

Whilst implementation of genomic data into epidemiological models provides the most detailed understanding of nosocomial transmission networks, the primary limitation of using WGS data during a pandemic when case numbers are high is that not all samples can be sequenced. In this case, we generated sequence data for 64.4% of the total number of positive cases (**Figure 1A**), suggesting that links within transmission networks may have been missing from our data set. In addition, whilst routine swabbing of staff and patients will have identified some asymptomatic cases, it is likely that other links in the chain may have been missed. Despite this, our data represent a robust snapshot of SARS-CoV-2 transmission at QAH over the second wave of COVID-19 infections in the UK and provide a powerful resource for understanding nosocomial transmission. In particular, these data highlight that high community prevalence can result in large scale increases in cases, and nosocomial spread of all circulating variants as resources become stretched.

It was interesting to note from our investigation that many of the wards affected by outbreaks were older hospital areas with poorer ventilation and infrastructure than more modern areas, which may play a significant role in transmission. The patient admissions pathway introduced at PHU in May 2020 and updated in May 2021 (**Supplementary Figure 2**) also recommended guidelines that prioritised use of wards in the modern estate (circa 2000s) with access to mechanical ventilation, particularly for infectious elderly patients.

Finally, the role of super-spreaders in nosocomial infections is also highlighted in our data, suggesting that rapid approaches to identifying and limiting interactions with such patients represents a key step in managing infection control. Indeed, the introduction of POCT for all patients in the emergency department in February 2021 allowed for rapid identification and cohorting of COVID-19 positive patients from admission. This appears to have had a major impact in reducing spread of the virus, although this was introduced later in the Alpha wave when case numbers were already in decline and where vaccine programs were underway, so the precise impact is difficult to define.

During the first wave of infections in the UK, many patients deemed medically fit were discharged to the community, and elective work typically performed at Queen Alexandra Hospital was ceased or moved to alternative clinical sites. Only acute medicine, acute surgery and cancer care continued throughout. Conversely, during the period discussed in this study, between September 2020 and May 2021, much elective surgery and non-acute care had reopened (with screening as outlined in **Supplementary Figure 2**). There were no large shifts in policy over this period that would have significantly impacted on the population of patients within the hospital, although care of those with COVID-19 infection often took priority over elective admissions. In addition, patient behaviour in seeking medical assistance meant people were often sicker when they presented, as people avoided attending for fear of infection with COVID-19. These factors meant that when cases peaked in the Alpha wave, beds became prioritised for COVID-19 patients and resources were moved from theatres to support ICU, allowing an increase from 20 funded ICU beds to 61 ICU available beds, providing 320% capacity (for both COVID-19 and non-COVID-19 patients). This response to the acute pressures of severe cases of COVID-19 throughout the Alpha wave, along with additional requirements for isolation of patients otherwise fit for discharge, may thus have had an indirect impact on the patient population and hospitalisation times utilised in this study. So whilst hospital policy remained largely unchanged over this time (**Supplementary Figure 1**), the patient population may have varied in response to COVID-19 pressures and clinical needs.

Transmission network analyses combining WGS genomic and epidemiological approaches, developed and expanded throughout the COVID-19 pandemic, will have benefits in years to come in providing accurate information on nosocomial outbreaks with other pathogens (Flowers et al., 2022). Furthermore, linking these data to patient outcomes will allow us to understand the role of nosocomial spread in severe disease, an element which we aim to address in further research. Future pandemics of the scale seen for COVID-19 will require WGS resources and capacities to be substantially scaled up within hospital settings or institutes closely linked to hospitals. This facility will allow a combined epidemiological and genomic analysis method to reach its full potential in aiding both immediate infection control and understanding of pathogen transmission dynamics at the research level.

## Data availability statement

The datasets presented in this study can be found in online repositories. The names of the repository/repositories and accession number(s) can be found in the article/**Supplementary Material**.

## Ethics statement

The studies involving human participants were reviewed and approved by the Health Research Authority (HRA) and Health and Care Research Wales (HCRW) following a favorable opinion from the North West – Haydock Research Ethics Committee on 24th April 2020 (Ref: 20/NW/0217). Participants were offered the opportunity to opt-out of having their anonymized data used in this study retrospectively. This work is part of the Sequencing and Tracking of Phylogeny (STOP COVID-19) study, which was posted to ClinicalTrials.gov (Ref: NCT04359849) on 24th April 2020. This work also forms part of the wider COVID-19 Genomics UK (COG-UK) Consortium surveillance study, which was approved by the Public Health England Research Ethics Governance Group and granted ethical approval by the PHE Research Ethics and Governance Group (REGG) on 8th April 2020, (PHE R&D ref: R&D NR0195). Written informed consent from the participants’ legal guardian/next of kin was not required to participate in this study in accordance with the national legislation and the institutional requirements.

## Author contributions

The manuscript was prepared by KC and SR with contributions from all authors. SR led the south coast COG-UK sequencing team, with assistance from SGl and AB. SR and AC conceived of the study and obtained funding. SR, KC, and KL performed bioinformatics analyses of genomic data. AB, SGl, SGo, CF, KC, KL, and SE worked on sequencing of SARS-CoV-2 genomes within the COG-UK study. SW, AL, and KB led SARS-CoV-2 clinical testing at QAH and provided samples for sequencing for COG-UK. SL and AC provided access to clinical data. All authors have approved the final manuscript.
