# Supplementary material for: Multiple pathways of SARS-CoV-2 nosocomial transmission uncovered by integrated genomic and epidemiological analyses during the second wave of the COVID-19 pandemic in the UK
Source: Front Cell Infect Microbiol. 2023 Jan 20;12:1066390. doi: 10.3389/fcimb.2022.1066390 (PMC9895378; doi:10.3389/fcimb.2022.1066390)
Supplement: Supplementary file 1 [file DataSheet_1.zip › SupplementaryInformation/SupplementaryFile.docx]

**Supplementary Material**

**Supplementary Figure 1:** Timeline of key actions taken by the Infection Prevention & Control (IPC) Team at Portsmouth Hospitals University NHS Trust (PHU) across the course of the pandemic, up to 1^st^ June 2021. The number of COVID-19 cases identified (7 day rolling average) is shown for all cases within PHU (blue line), and for community cases within Hampshire (red line). The period studied in this research paper is highlighted in grey. Case numbers were taken from <https://coronavirus.data.gov.uk> (accessed 6^th^ December 2022). PPE, Personal Protective Equipment; LFT, Lateral Flow Test; HCW, Health Care Worker; POCT, Point of Care test; ED, Emergency Department.


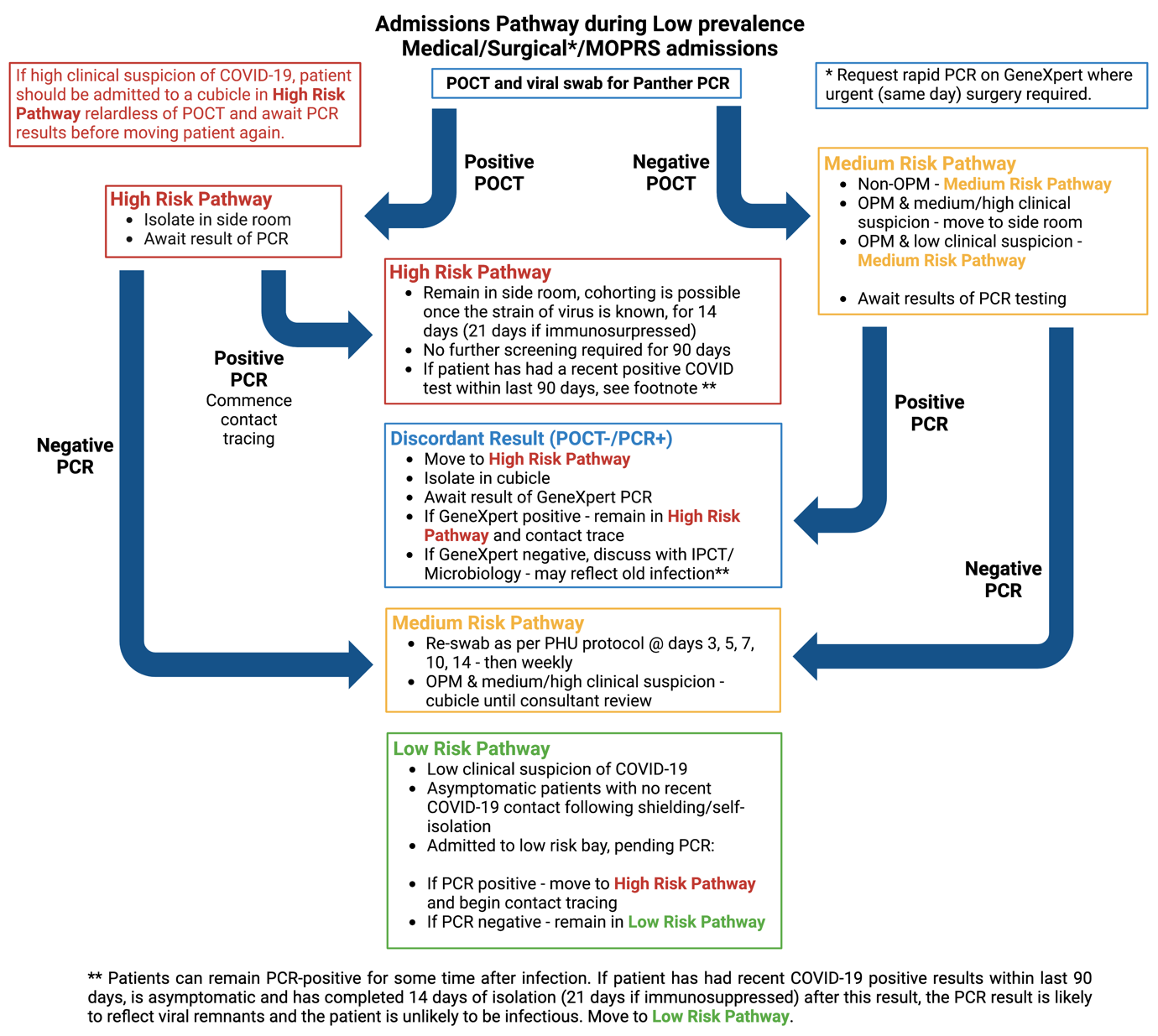


**Supplementary Figure 2:** Outline of the guidance for the safe movement of patients into and across the Trust during the COVID-19 pandemic. This pathway was introduced in May 2020, and updated regularly following local and national changes in guidelines. This figure represents the updates as of May 2021. “Low prevalence” is based on the PHE definition of less than 0.5% population prevalence. All renal, hematology and oncology patients received rapid PCR (GeneXpert) on admission and were isolated pending results. Direct admissions from the Emergency Department to the Department of Critical Care required POCT and rapid PCR. POCT was provided using the Abbott ID Now SARS-CoV-2, PCR testing was performed using the Hologic Panther system, and rapid PCR was performed using the GeneXpert assay. MOPRS, Medicine for Older Persons; POCT, Point of Care Test; PCR, Polymerase Chain Reaction; OPM, Older People’s Medicine; IPCT, Infection Prevention & Control Team. Created with BioRender.com.

**
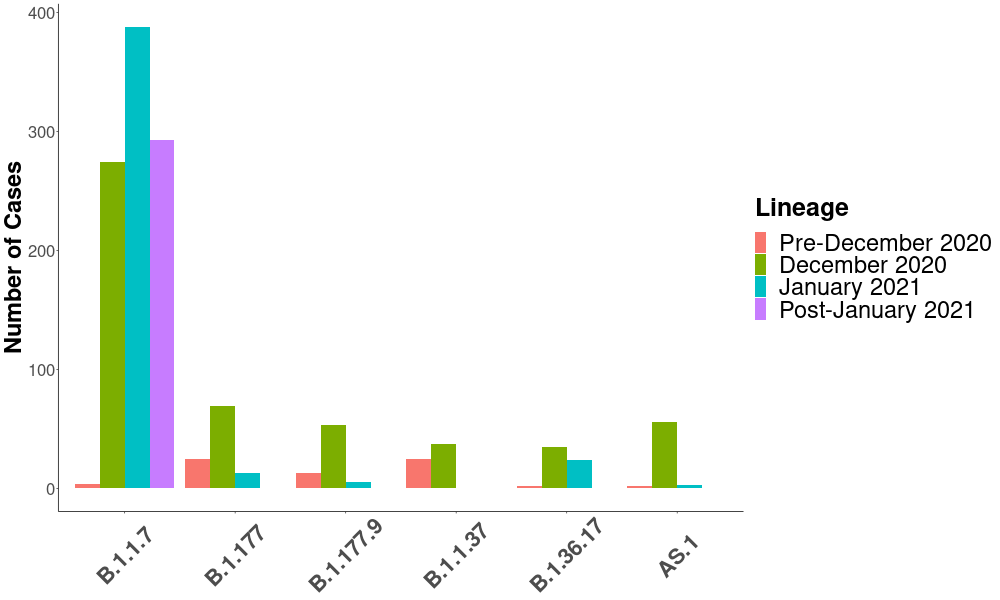
**

**Supplementary Figure 3:** Change in case numbers of circulating SARS-CoV-2 variants over winter 2020/2021. Case numbers are shown for September 2020 until November 2020 ('Pre-December 2020'), December 2020, January 2021, and February 2021 until May 2021 ('Post-January 2021'). The introduction of the Alpha variant B.1.1.7 in December 2020 resulted in substantial increase in all circulating variants, but B.1.1.7 became the sole circulating variant by February 2021.

**
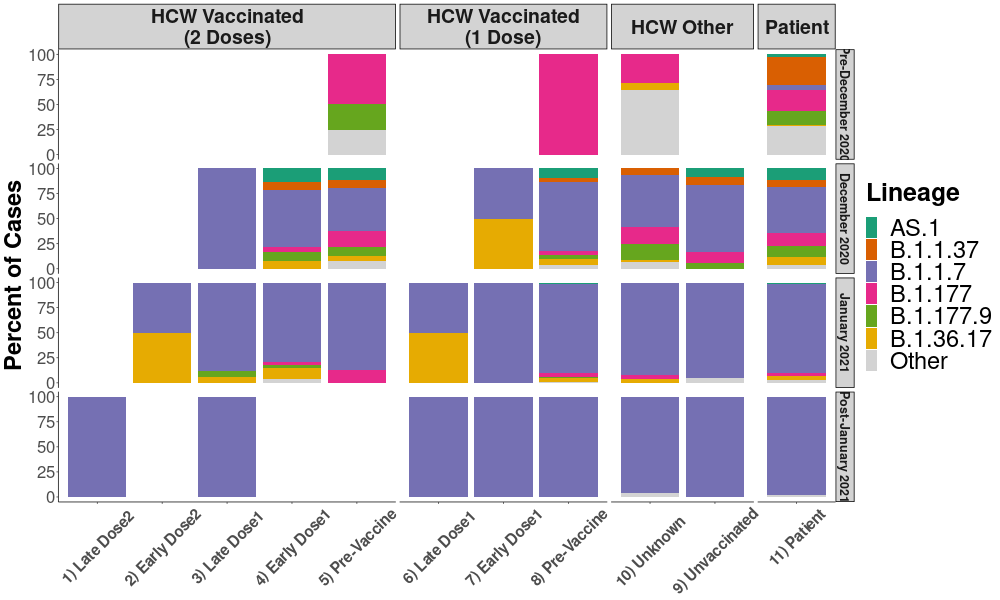
**

**Supplementary Figure 4:** Distribution of lineages identified amongst SARS-CoV-2 samples, for four distinct time periods (September to November 2020 , December 2020, January 2021 and February to May 2021), for 1) HCWs with 2 doses testing positive 15 days or longer from the second vaccine dose (Late Dose2), 2) HCWs with 2 doses testing positive 14 days or fewer from the second vaccine dose (Early Dose2), 3) HCWs with 2 doses testing positive 15 days or longer from the first vaccine dose (Late Dose1), 4) HCWs with 2 doses testing positive 14 days or fewer from the first vaccine dose (Early Dose1), 5) HCWs with 2 doses testing positive prior to vaccination, 6) HCWs with 1 dose testing positive 15 days or longer from the first vaccine dose (Late Dose1), 7) HCWs with 1 dose testing positive 14 days or fewer from the first vaccine dose (Early Dose1), 8) HCWs with 1 dose testing positive prior to vaccination, 9) non-vaccinated healthcare workers (HCWs), 10) HCWs with an unknown vaccination status (assumed non-vaccinated), or 11) patients within the hospital (primarily unvaccinated at this time). c) Distribution of distinct lineages identified on a weekly basis at QAH. Distinct lineages are identified based on the Pangolin tool. Lineages with fewer than 5 cases are combined into a single class ('Other'). Case numbers are here based on sequencing results only, so represent a subset of the total cases observed at QAH over this time.

**Supplementary Table 1: Accession IDs for SARS-CoV-2 data utilised in this study.** This table contains the unique COVID-19 Genomics UK (COG-UK) Consortium ID assigned to each sample (central_sample_id), Global Initiative for Sharing of All Influenza Data (GISAID) accession IDs (gisaid.accession and gisaid.secondary_accession), and European Nucleotide Archive (ENA) where data are available under accession PRJEB37886 at the sample (ena_sample.accession, ena_sample.secondary_accession), run (ena_run.accession), and consensus assembly (ena_assembly.accession) level.

**Supplementary Data: Full list and affiliations for COVID-19 Genomics UK (COG-UK) Consortium members.**
