# Supplementary figures and images for: Multiple pathways of SARS-CoV-2 nosocomial transmission uncovered by integrated genomic and epidemiological analyses during the second wave of the COVID-19 pandemic in the UK
Source: Front Cell Infect Microbiol. 2023 Jan 20;12:1066390. doi: 10.3389/fcimb.2022.1066390 (PMC9895378; doi:10.3389/fcimb.2022.1066390)

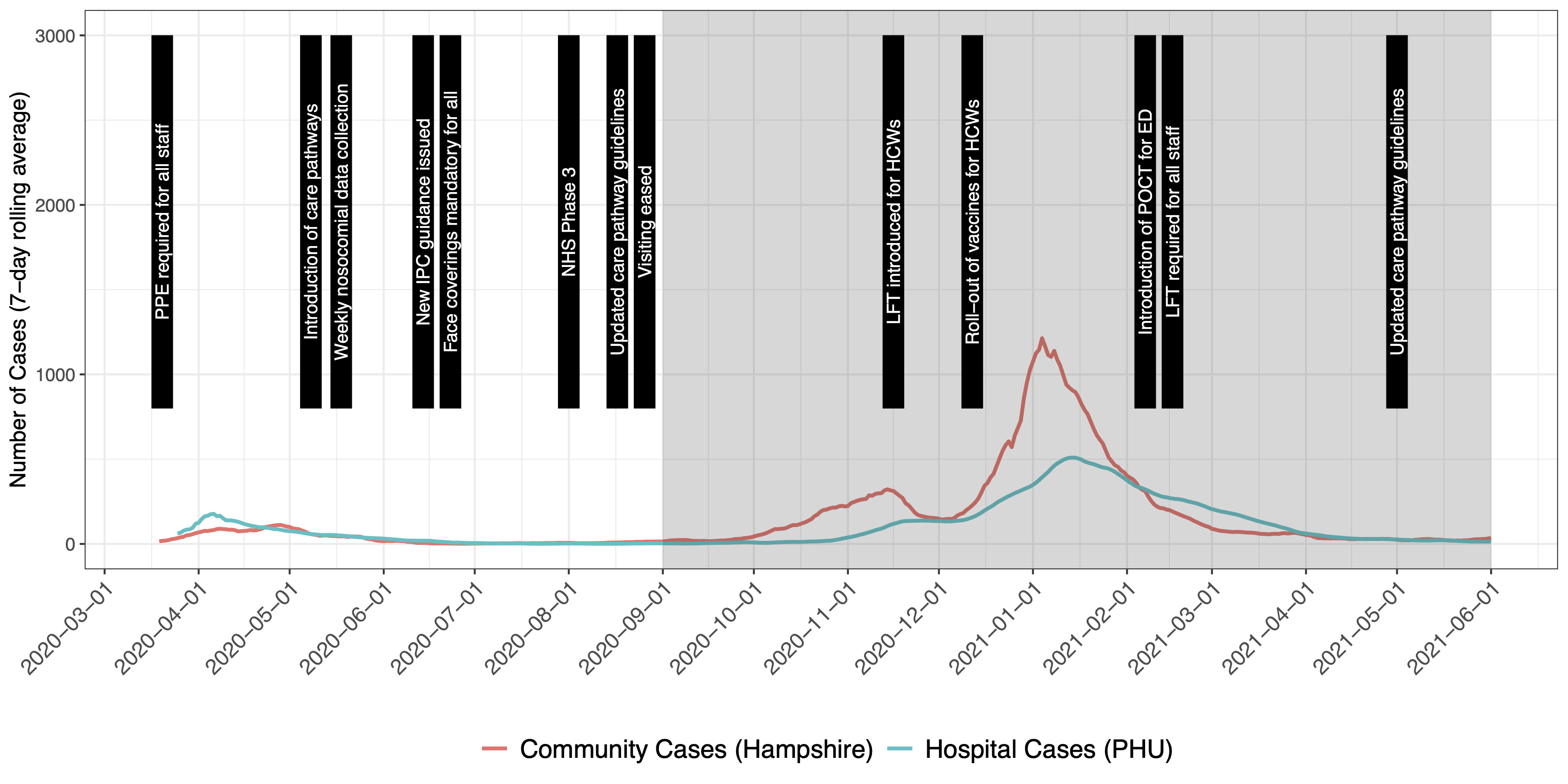

Supplement: Supplementary file 1 [file DataSheet_1.zip › SupplementaryInformation/SupplementaryFigure1.tiff]

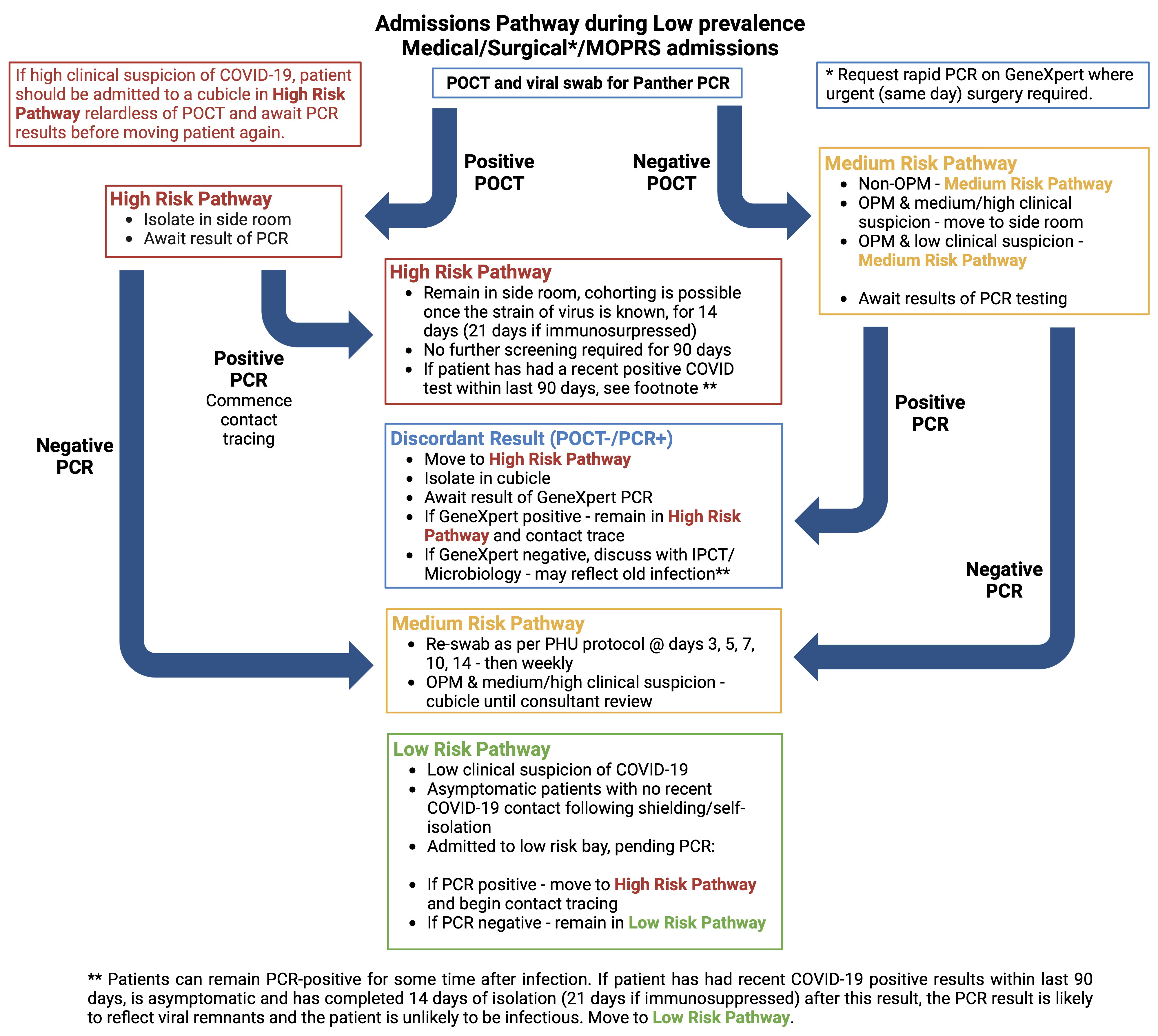

Supplement: Supplementary file 1 [file DataSheet_1.zip › SupplementaryInformation/SupplementaryFigure2.tiff]
